# Targeted creation of new mutants with compact plant architecture using CRISPR/Cas9 genome editing by an optimized genetic transformation procedure in cucurbit plants

**DOI:** 10.1093/hr/uhab086

**Published:** 2022-01-20

**Authors:** Tongxu Xin, Haojie Tian, Yalin Ma, Shenhao Wang, Li Yang, Xutong Li, Mengzhuo Zhang, Chen Chen, Huaisong Wang, Haizhen Li, Jieting Xu, Sanwen Huang, Xueyong Yang

**Affiliations:** Key Laboratory of Biology and Genetic Improvement of Horticultural Crops of Ministry of Agriculture, Sino-Dutch Joint Lab of Horticultural Genomics, Institute of Vegetables and Flowers, Chinese Academy of Agricultural Sciences, Beijing 100081, China; Key Laboratory of Biology and Genetic Improvement of Horticultural Crops of Ministry of Agriculture, Sino-Dutch Joint Lab of Horticultural Genomics, Institute of Vegetables and Flowers, Chinese Academy of Agricultural Sciences, Beijing 100081, China; Key Laboratory of Biology and Genetic Improvement of Horticultural Crops of Ministry of Agriculture, Sino-Dutch Joint Lab of Horticultural Genomics, Institute of Vegetables and Flowers, Chinese Academy of Agricultural Sciences, Beijing 100081, China; Agricultural Genomics Institute at Shenzhen, Chinese Academy of Agricultural Sciences, Shenzhen 518124, China; College of Horticulture, Northwest A&F University, Yangling 712100, China; Key Laboratory of Horticultural Plant Biology, Ministry of Education, College of Horticulture and Forestry Sciences, Huazhong Agricultural University, Wuhan 430070, China; Key Laboratory of Biology and Genetic Improvement of Horticultural Crops of Ministry of Agriculture, Sino-Dutch Joint Lab of Horticultural Genomics, Institute of Vegetables and Flowers, Chinese Academy of Agricultural Sciences, Beijing 100081, China; Key Laboratory of Biology and Genetic Improvement of Horticultural Crops of Ministry of Agriculture, Sino-Dutch Joint Lab of Horticultural Genomics, Institute of Vegetables and Flowers, Chinese Academy of Agricultural Sciences, Beijing 100081, China; Agricultural Genomics Institute at Shenzhen, Chinese Academy of Agricultural Sciences, Shenzhen 518124, China; Hunan Vegetable Research Institute, Hunan Academy of Agricultural Sciences, Changsha 410125, China; Key Laboratory of Biology and Genetic Improvement of Horticultural Crops of Ministry of Agriculture, Sino-Dutch Joint Lab of Horticultural Genomics, Institute of Vegetables and Flowers, Chinese Academy of Agricultural Sciences, Beijing 100081, China; Beijing Vegetable Research Institute, Beijing Academy of Agriculture and Forestry Sciences, Beijing, 100097, China; Wimi Biotechnology (Jiangsu) Co., Ltd, Changzhou, 213000, China; Agricultural Genomics Institute at Shenzhen, Chinese Academy of Agricultural Sciences, Shenzhen 518124, China; Key Laboratory of Biology and Genetic Improvement of Horticultural Crops of Ministry of Agriculture, Sino-Dutch Joint Lab of Horticultural Genomics, Institute of Vegetables and Flowers, Chinese Academy of Agricultural Sciences, Beijing 100081, China

## Abstract

Fruits and vegetables in the *Cucurbitaceae* family, such as cucumber, melon, watermelon, and squash, contribute greatly to the human diet. The widespread use of genome editing technologies has greatly accelerated gene functional characterization and crop improvement. However, most economically important cucurbit plants, including melon and squash, remain recalcitrant to standard *Agrobacterium tumefaciens*-mediated transformation, limiting the effective use of genome editing technology. In this study, we used an “optimal infiltration intensity” strategy to establish an efficient genetic transformation system for melon and squash. We harnessed the power of this method to target homologs of the *ERECTA* family of receptor kinase genes and created alleles that resulted in a compact plant architecture with shorter internodes in melon, squash, and cucumber. The optimized transformation method presented here enables stable CRISPR/Cas9-mediated mutagenesis and provides a solid foundation for functional gene manipulation in cucurbit crops.

## Introduction

The *Cucurbitaceae* family is one of the most genetically diverse crops worldwide [1]. Cultivation of cucurbit crops occupies 2.6% of the total global arable land devoted to vegetable crops. Cucurbits include various vegetables and fruits, of which melon (*Cucumis melo* L.), squash (*Cucurbita moschata* Duchesne), watermelon (*Citrullus lanatus*), and cucumber (*Cucumis sativus* L.) are the four most widely cultivated [2] and most popular species, according to the Food and Agriculture Organization (FAO) of the United Nations. Since the 1970s, production of these crops has increased by 483%, and there remains potential for further increases in production and quality improvement through traditional breeding and biotechnological means.

The manipulation of gene expression or function and biotechnological advances have greatly empowered the molecular characterization of functional genes in cucurbit crops and will inform new methods of breeding cucurbit species for economically important traits [3]. In particular, the CRISPR (Clustered Regularly Interspaced Short Palindromic Repeat) and CRISPR-associated nuclease 9 (Cas9) system is a powerful tool for reaching this goal. The CRISPR/Cas9 system comprises a single guide RNA (sgRNA) molecule with a specific target sequence that is complementary to a gene of interest, and the DNA endonuclease Cas9 [4–6]. The application of the CRISPR/Cas9 system requires a stable and universal method for delivering the CRISPR/Cas9 reagents into plant cells [7]. The common delivery methods include protoplast transfection, *Agrobacterium* (*Agrobacterium tumefaciens*)-mediated transformation, or particle bombardment [8]. *Agrobacterium*-mediated transformation is the most widely used delivery method for generating transgenic plants and producing genome-edited germplasm. Successful CRISPR/Cas9-mediated genome editing has been reported in the model plant Arabidopsis (*Arabidopsis thaliana*) and in an increasing number of important crops, such as rice (*Oryza sativa*), sorghum (*Sorghum bicolor*), wheat (*Triticum aestivum*), tomato (*Solanum lycopersicum*), grape (*Vitis vinifera*), and maize (*Zea mays*) [9–11]. Although cucumber has been successfully genetically transformed and genome edited [12, 13], transformation efficiencies remain very low. For watermelon, although a transformation method and genome editing have developed rapidly in recent years, this transformation method uses indirect organogenesis for regeneration [14], which is currently not compatible with other cucurbit crops. Therefore, most other cucurbit plants of economic value, such as melon and squash, remain recalcitrant to standard *Agrobacterium*-mediated transformation, thus limiting the rapid application of genome editing and functional gene research to these crops. To our knowledge, there have been no reports of heritable genome editing in melon and squash.

The efficiency of plant genetic transformation is influenced by different factors, including the regeneration pathway, the method of infection, the type of starting explant, the *Agrobacterium* strain, and the culture medium [15–17]. Plant regeneration relies on the principle of cell totipotency, whereby one or more plant cells can be directed to regenerate an entire plant via somatic embryogenesis and organogenesis [18, 19]. During somatic embryogenesis, totipotent cells can form somatic embryos and then regenerate entire plants following the embryogenic pathway. Organogenesis refers to the formation of organs from cultured explants and may involve both direct and indirect methods. In the case of direct organogenesis, the formation of new organs is directly induced from explant tissues, whereas in indirect organogenesis, organs are formed *de novo* from an intermediate tissue, the callus [20]. Although each method has advantages and disadvantages, direct organogenesis is typically suitable for horticultural crops because it is less prone to spontaneous mutation and can be implemented in a shorter time frame.

Infiltration with an *Agrobacterium* cell suspension is the most decisive factor for genetic transformation when opting for direct organogenesis [21]. Many reports have improved transformation efficiency to a certain extent by increasing the infection intensity [13, 22–24], but there are currently no infection standards concerning the most appropriate infection intensity for more efficient transformation. Variable infection may lead to unreliable results: if there is insufficient infection, regeneration-competent cells will not be infected, whereas excessive infection may cause severe explant damage. Previous studies have shown that adventitious shoots can initiate from procambium or cambium cells in different plant species during direct organogenesis [25]. As procambium or cambium cells are embedded in the deeper vascular layers, we speculated that effective infiltration of the vascular cells would be a critical step toward efficient transformation.

To develop a more efficient and reproducible genetic transformation method via direct organogenesis and achieve heritable genome editing in major cucurbit crops, we propose an “optimal infiltration intensity” strategy. In this strategy, cotyledonary node explants are wounded with a micro-brush and sonicated according to optimized parameters of infiltration conditions. Regeneration of entire plants via the direct organogenesis pathway then follows. This strategy succeeded in infecting cambium cells in the vascular tissue, which could be effectively induced into adventitious buds, while causing minimal explant damage. We successfully established a genetic transformation system for melon and squash using this strategy and improved the transformation efficiency of cucumber. We targeted the *Arabidopsis ERECTA* family of receptor kinase gene homologs in melon, squash, and cucumber [26, 27]. The resulting CRISPR/Cas9-mediated mutations were stably inherited and constitute the first example of heritable genome editing in melon and squash. These recessive mutant alleles produced shorter internodes, a phenotype that is useful in breeding.

## Results

### The optimal infiltration intensity strategy for genetic transformation of melon

**Figure 1:**
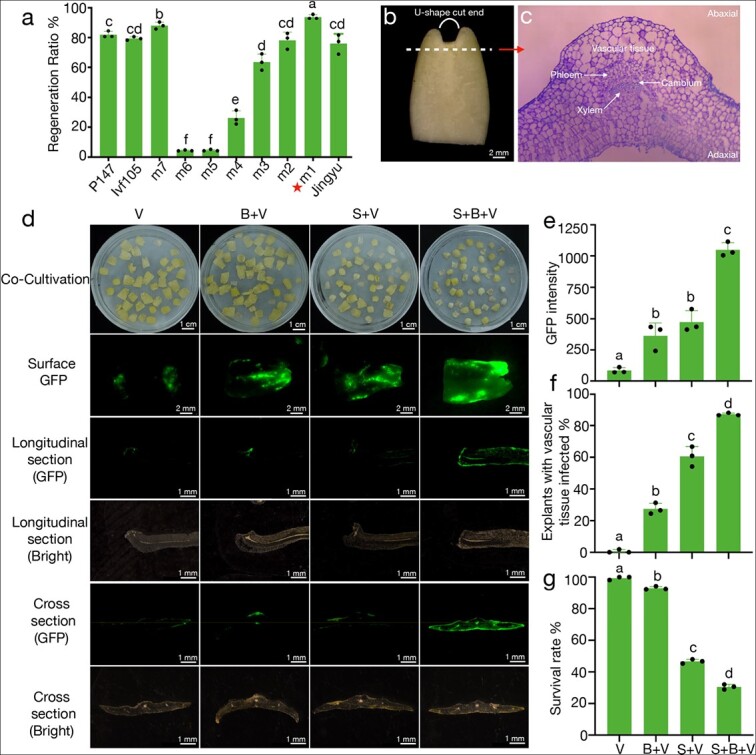
**Increasing infection intensity is the key to genetic transformation of melon.** (a) Shoot regeneration ratio of ten different melon varieties. For each variety, the regeneration ratio was calculated as the percentage of explants with regenerated shoots/total number of explants. The red star indicates that the highest rate was 93.8% for m1. Data are means of three replicates, and error bars indicate standard deviations. Different lowercase letters indicate significant differences (*p* < 0.05, Tukey’s test). (b) The cotyledon explant of melon. The curved white line represents the U-shaped cut end. The white dash represents the cross section of panel c. (c) Cross-section along the white dash of panel b, showing that the cambium cells are located in the deeper layer of vascular tissue. (d) From left to right, each column represents different infection treatments and effects. V: vacuum, B: micro-brush, S: sonication. The first line shows the cotyledon explants of melon after co-cultivation with *Agrobacterium tumefaciens* in the dark. From the second line to the sixth line, examination of GFP fluorescence after co-cultivation showed the region and intensity of the fluorescent signal. The third and fourth lines show the infected areas in longitudinal section. The fifth and sixth lines show the infected areas in cross-section. (e, f, and g) GFP intensity, explants with infected vascular tissue, and survival rate under four different treatments, respectively. Data are means of three replicates, and error bars indicate standard deviations. Different lowercase letters indicate significant differences (*p* < 0.05, Tukey’s test).

Stable and highly efficient regeneration via direct organogenesis is the basis of genetic transformation. To establish a genetic transformation system for melon, we first tested ten melon varieties to identify those with higher regeneration efficiency. For each variety, 80–110 explants were cultured in regeneration medium for about one month with three biological replicates. The regeneration rate was calculated as the percentage of explants with regenerated shoots divided by the total number of explants. Three of the cultivars had a regeneration efficiency greater than 80%. Among these, the cultivar “m1” had the highest regeneration efficiency (93.8%) and was therefore used for further investigations (Figure 1a and Table S1).

Procambium and cambium contain the initial target cells for regeneration are located in the deeper layers of the vascular tissue (Figure 1b and 1c). We first adopted a previously reported green fluorescent protein (GFP)-based system [13] to evaluate the effect of infection by vacuum infiltration on cultivar m1. Vascular tissue located in this deep layer was difficult to infect, suggesting that most explants were insufficiently infected by this method (Figure S1a–d). To improve the infection intensity, we increased the duration of vacuum infiltration, but we observed higher GFP fluorescence only on the surface of the explants and not deep in the vascular tissue (Figure S1e–f). Importantly, a longer duration of infiltration also led to severe explant damage and over-proliferation of *Agrobacteria* (Figure S1g).

To facilitate *Agrobacterium* infection of vascular tissues, we used sonication and micro-brush (KITA, Nanotek Brush) treatments based on vacuum infiltration to increase the infection rate. Sonication produced numerous micro-wounds, mainly on the surface, but also sometimes reaching deeper into the explant tissues [28–32]. Scratching the cotyledon nodes using a micro-brush resulted in uniform and deep wounding [33]. Both methods have been shown to increase infection intensity and transformation efficiency [34–36]. Accordingly, we first compared the effects of micro-brushing and sonication alone or in combination on *Agrobacterium*-mediated transformation.

**Figure 2:**
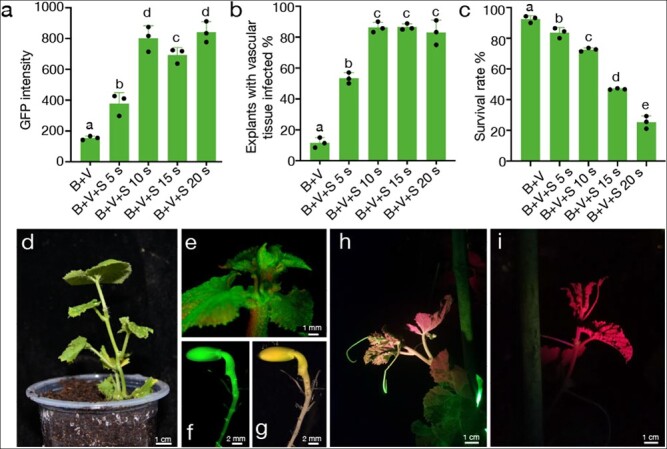
**The genetic transformation system of melon was established using the strategy of optimal infiltration intensity.** (a, b and c) GFP intensity, explants with infected vascular tissue, and survival rate of melon m1 under five different treatments, respectively. Data are means of three replicates, and error bars indicate standard deviations. Different lowercase letters indicate significant differences (*p* < 0.05, Tukey’s test). (d) A regenerated transgenic T_0_ plant of melon was transferred to the soil. The GFP-fluorescent apical meristem (e) and seeds (f and g) of the transgenic melon T_0_ plant. The transgenic T_1_ plant (h) and WT (i) of melon under the GFP channel of the LUYOR-3415RG. Tissues appear green because of GFP expression and red because of chlorophyll autofluorescence.

We exposed explants to four treatments: 1) vacuum infiltration alone (V); 2) scratching the proximal regions of explants with a micro-brush, followed by vacuum infiltration (B + V); 3) sonication for 20 s, followed by vacuum infiltration (S + V); and 4) a combination of micro-brushing with sonication, followed by vacuum infiltration (S + B + V). Vacuum infiltration alone (V) yielded relatively low GFP fluorescence in the infected explants, with no evidence of fluorescence in the deep layers of the vascular tissues, indicating that they were not infected (Figure 1d and 1e). Scratching the explants with a micro-brush (B + V) produced regular wounds and increased GFP fluorescence along the proximal regions of explants. However, microscopic observations showed that use of the micro-brush alone had a limited effect on vascular tissue infection, suggesting that the vasculature may be out of reach of the micro-brush (Figure 1d). When we added sonication to the explant treatment, we observed considerable enhancement of GFP abundance on the surface of explants (Figure 1d and 1e). Notably, the combination of vacuum infiltration, sonication, and micro-brushing resulted in the strongest GFP abundance, with 87% of explants showing effective infection in the vascular tissues (Figure 1d–g). We speculate that the surface and deeper wounds produced by micro-brushing probably greatly increased the chances that sonication would make deeper wounds that reached the vascular explant tissue. However, although the combination of vacuum infiltration, micro-brushing, and sonication increased the infection rate, explants also exhibited severe tissue disruption and frequently died, directly affecting the subsequent regeneration of explants (Figure 1g, Figure S1h–k).

Sonication had the greatest influence on explant growth (Figure 1g). Therefore, to further optimize and establish optimal infiltration intensity conditions, we treated explants with varying durations of sonication from 0 to 20 s. Different infection intensities had different effects on the growth state of the cotyledon explants. Explants infiltrated with *Agrobacterium* but not exposed to sonication displayed healthy growth after co-cultivation, whereas increasing the duration of sonication clearly negatively affected explant survival rate. For instance, approximately 70% of all explants survived when sonicated for 10 s, but the survival rate fell below 40% when sonication was longer than 10 s, as evidenced by the curling of explants and the over-proliferation of *Agrobacteria* (Figure 2c, Figure S2). The duration of sonication also had a notable effect on the abundance of GFP in explants (Figure 2a, Figure S2). Compared with controls (B + V), explants exposed to sonication showed increases in GFP fluorescence and the proportion of explants with infected vascular tissue (Figure 2a–2c, Figure S2). The GFP abundance increased with increasing duration of sonication from 5 s to 20 s. We obtained a higher level of GFP fluorescence with sonication for 10 s, resulting in 86% of explants with infected vascular tissues, but longer sonication durations did not further improve the infection rate (Figure 2a–c). In summary, when micro-brushing, 10 s sonication, and vacuum infiltration of explants were combined, the vascular tissue of up to 86% of all explants was infected without negative effects on subsequent regeneration. We named these optimized conditions the optimal infiltration intensity.

To evaluate the effects of optimal infiltration intensity on transformation efficiency, we scored the number of GFP-positive T_0_ plants (Figure 2d and 2e) and divided this number by the number of surviving explants. We obtained a transformation efficiency of 3.17% for melon m1 (Table 1), demonstrating that the optimal infiltration intensity strategy remarkably improves the transformation efficiency of melon. Transformed genes can also be stably inherited to the T_1_ generation through pollination (Figure 2f–i).

**Table 1:** Transformation efficiency of melon m1 infection intensity treatments

**Treatment**	**No. of explants** ^**a**^	**No. of GFP-positive T**_**0**_ **plants**	**Transformation efficiency (%)**	**Significance** ^**b**^
B + V	508	0	0.0 ± 0.0	c
B + V + S5s	440	6	1.32 ± 0.30	b
B + V + S10s	472	15	3.17 ± 0.13	a
B + V + S15s	391	3	1.02 ± 0.24	b
B + V + S20s	372	1	0.24 ± 0.24	c

Data for transformation efficiency are means ± SEM of three independent trials. ^a^No. of explants is the sum of explants that survived after infection in three independent trials. ^b^Different letters (a, b, and c) indicate significant differences among treatments (*p* < 0.05, Tukey’s test).

**Figure 3:**
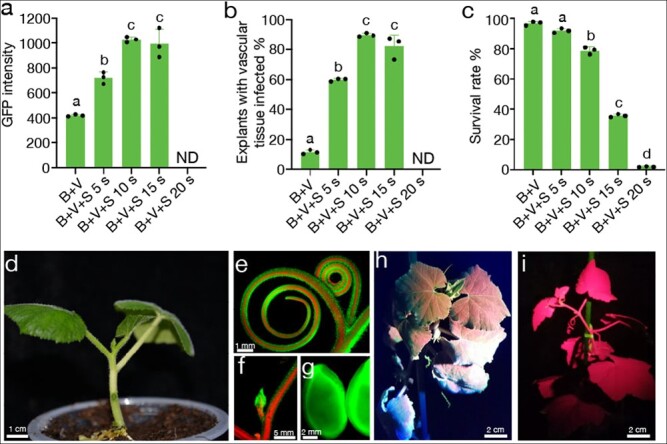
**The genetic transformation system of squash was established using the strategy of optimal infiltration intensity.** (a, b and c) GFP intensity, explants with infected vascular tissue, and survival rate of squash jingxinzhen No. 4 under five different treatments, respectively. Data are means of three replicates, and error bars indicate standard deviations. Different lowercase letters indicate significant differences (*p* < 0.05, Tukey’s test). (d) A regenerated transgenic T_0_ plant of squash after one week of transfer to soil. The GFP-fluorescent tendril (e), apical meristem (f), and seeds (g) of a transgenic squash T_0_ plant. A transgenic T_1_ plant (h) and WT plant (i) of squash under the GFP channel of the LUYOR-3415RG.

### The optimal infiltration intensity strategy and antioxidant application for genetic transformation of squash

Based on the success of the optimal infiltration intensity strategy in melon, we next tested whether this strategy was compatible with squash. We selected two commercial squash varieties, “jingxinzhen No. 4” and “jingxinzhen No. 11” with high regeneration efficiency (87.6% and 78.4%, respectively) (Figure S3a–S3c). Explants of “jingxinzhen No. 4” and “jingxinzhen No. 11” were exposed to sonication ranging in duration from 0 to 20 s in combination with micro-brushing and vacuum infiltration of *Agrobacterium*. We observed a high mortality rate among explants after sonication, even at a duration of only 5 s (Figure S3d and S3e). This result underscores the low tolerance of this cultivar to wounding. *Agrobacterium*-mediated infection is well known to impose stress on plant cells, thus affecting plant tissue survival and regeneration. Several antioxidants, such as silver nitrate (AgNO_3_), cysteine (Cys), dithiothreitol (DTT), polyvinylpyrrolidone (PVP), and lipoic acid (LA) can reduce browning and death of plant tissues when added to the culture medium [37–40].

LA is a sulfur-containing substance involved in several multi-enzyme complexes that can prevent the oxidation of metabolic compounds in cells and minimize the extent of tissue disruption [41]. LA also passes easily through the permeable cell wall, improves the survival rate of transformed cells after explant infection with *Agrobacterium*, and significantly improves the transformation efficiency of some recalcitrant plant species [42]. We therefore tested the effects on explants of adding 250 μM LA to the co-culture medium. Indeed, explants of jingxinzhen No. 4 and jingxinzhen No. 11 showed a significant improvement in survival following sonication for 5 s, micro-brushing, and vacuum infiltration of *Agrobacterium* in the presence of LA (Figure S3f and S3g). A gradient infection intensity test was then performed on jingxinzhen No. 4 with the addition of LA. The optimal infiltration intensity comprised micro-brushing and sonication for 10 s, followed by vacuum infiltration. This resulted in the infection of cambium cells in 90% of the explants, with no adverse effects on survival rate or subsequent regeneration of transgenic plants (Figure 3a–3c, Figure S4), and yielded a final transformation efficiency of 3.56% (Table 2). GFP fluorescence was detected in all tissues of T_0_ squash plants, including apical meristems, tendrils, and seeds (Figure 3d–3g). Importantly, the transgene was stably inherited to the T_1_ generation (Figure 3h and 3i). These results indicate that optimal infiltration intensity is amenable to the genetic transformation of melon and squash. Sufficiently increasing infection while ensuring that infected explants remain healthy is essential for their stable genetic transformation.

**Table 2:** Transformation efficiency of squash jingxinzhen No. 4 infection intensity treatments

**Treatment**	**No. of explants** ^**a**^	**No. of GFP-positive T**_**0**_ **plants**	**Transformation efficiency (%)**	**Significance** ^**b**^
B + V	642	0	0.0 ± 0.0	c
B + V + S5s	410	5	1.20 ± 0.20	b
B + V + S10s	392	14	3.56 ± 0.09	a
B + V + S15s	403	3	1.00 ± 0.22	b
B + V + S20s	374	0	0.0 ± 0.0	c

Data for transformation efficiency are means ± SEM of three independent trials. ^a^No. of explants is the sum of explants that survived after infection in three independent trials. ^b^Different letters (a, b, and c) indicate significant differences among treatments (*p* < 0.05, Tukey’s test).

Based on the encouraging level of explant protection observed with the addition of LA, we included LA in the optimal infiltration intensity strategy to further improve the transformation efficiency of melon m1. The addition of LA increased the transformation efficiency of melon m1 from 3.2% to 4.0% (Table 3). A gradient infiltration intensity experiment was also carried out in different types of cucumber using the same strategy. The optimal duration of sonication required to reach optimal infiltration intensity was 30 s for Cu2, Xintaimici, and 404, and 15 s for Eu1 (Figure S5a–5d). Under these optimized conditions, the genetic transformation of the four varieties was successful, and the efficiencies of F, Xintaimici, 404, and Eu1 were 5.18%, 2.20%, 1.97%, and 2.46%, respectively (Table 3). These results indicate that the optimal infiltration intensity strategy effectively improves the genetic transformation efficiency of cucumber and partially overcomes the classic issue of genotype dependence. This methodology may be compatible with other cucurbit crops, as well as other species.

**Table 3:** The antioxidant LA can further improve the transformation efficiency of different cucumber varieties and melon m1 under optimal infiltration intensity.

**Variety**	**No. of explants** ^**a**^	**No. of GFP-positive T**_**0**_ **plants**	**Transformation efficiency (%)** ^**b**^
m1	809	32	3.95
Cu2	2160	112	5.18
Xintaimici	636	14	2.20
404	254	5	1.97
Eu1	448	11	2.46

a No. of explants is the sum of multiple independent events. ^b^Data for transformation efficiency are the average of multiple independent events in different varieties.

**Figure 4:**
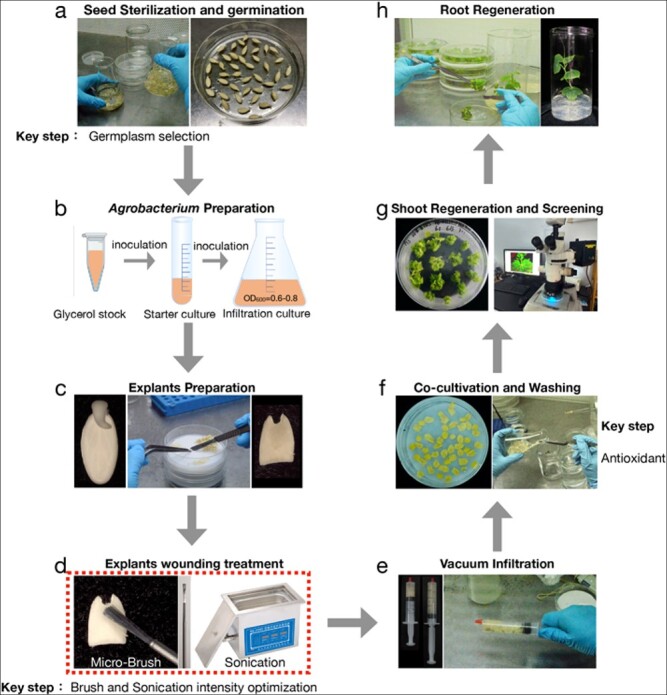
**Overview of the eight-stage procedure for *Agrobacterium*­mediated transformation.** (a) Removal of seed coat, sterilization, and germination. (b) Preparation of *Agrobacterium tumefaciens,* culturing in LB liquid medium, transfer, and shaking to OD = 0.6–0.8. (c) Explant preparation, excision of embryos from the germinated seeds, cutting of cotyledons in half transversely, and selection of proximal parts with U-shaped ends as explants. (d) Infection, scratching of the proximal regions of explants with a micro-brush, and sonication. (e) Vacuum infiltration. Vacuum treatment was applied with a syringe. (f) Co-cultivation with *A. tumefaciens* in the dark. (g) Shoot regeneration and screening. The dark-cultured explants were washed with sterilized water, then transferred to shoot induction medium. Explants regenerated for about 4 weeks and were then screened for positive buds by fluorescence microscopy. (h) Root regeneration. The positive buds were removed and transferred to rooting medium.

The detailed protocol for the optimal infiltration intensity strategy is illustrated in Figure 4. We concluded that the three key steps that should be explored for different varieties or species were the regeneration rate of germplasm, the optimal infiltration intensity, and use of the antioxidants during co-cultivation.

### CRISPR/Cas9-mediated editing of *ERECTA* (*ER*) gene homologs in cucurbit crops

CRISPR/Cas9-mediated genome editing is a powerful tool for genetic engineering and has been widely used in various crops. However, there are currently no reports on the heritable application of genome editing for melon or squash, as they are difficult to transform. One desirable trait for the cucurbit crops melon, squash, and cucumber in protected cultivation is a compact plant architecture and short internodes, which help to increase planting density and yield and save labor costs. Using the optimized transformation system described above, we examined the efficiency of CRISPR/Cas9-mediated genome editing by targeting cucurbit homologs to the *ERECTA* (*ER*) gene family. These genes encode receptor kinases known to control plant internode length in *Arabidopsis* [27].

**Figure 5:**
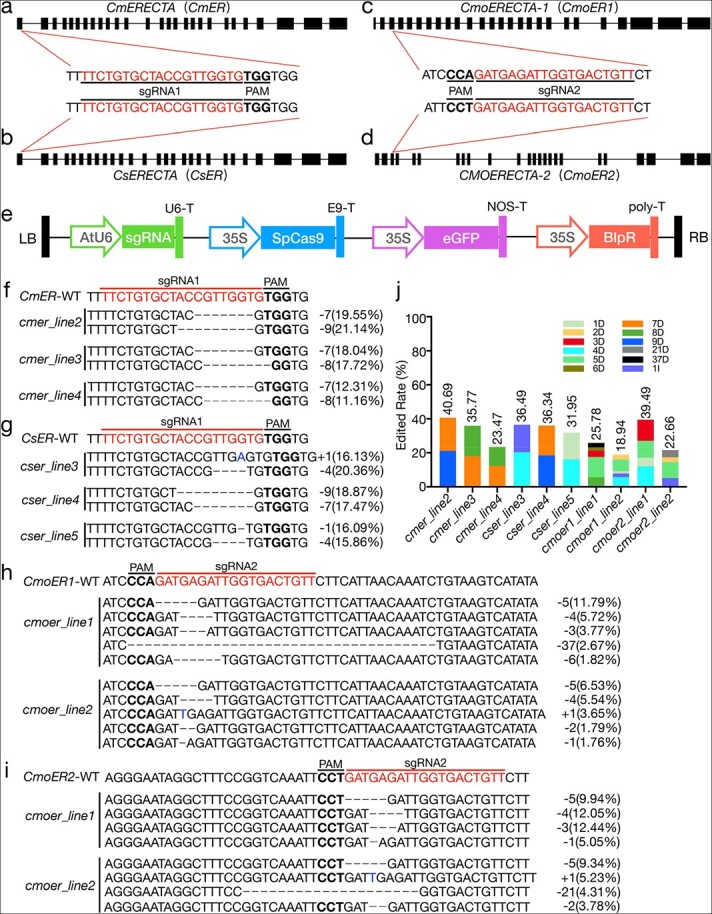
**Vector, sgRNA map, CRISPR/Cas9 editing target sites, and editing efficiency.** (a–d) Schematic view of sgRNA target sites and editing results for the *ER* gene in melon, cucumber, and squash. The target sequences are highlighted in red and underlined, and the PAM sites are highlighted in bold and underlined. (e) T-DNA region of the pBSE402 vector. (f–i) Genome editing results of T_0_ generation plants. sgRNA and protospacer-adjacent motif (PAM) sequences are highlighted by red and bold, respectively. Black dash indicates deletion. Blue letters indicate insertion. The text on the right indicates the editing type. The numbers in parentheses represent the proportion of this editing type in the total reads. (j) The columnar stacking diagram shows the target mutation rate based on Hi-TOM analysis (reads of target mutation/total reads of target site). Different colors represent different mutation types. D indicates deletion, and I indicates insertion.

We first queried the cucurbit genomes at the Cucurbit Genomics Database (CuGenDB; http://cucurbitgenomics.org) to identify putative homologs in melon (*MELO3C016916*), cucumber (*CsaV3_4G036080*), and squash (*CmoCh09G003660* and *CmoCh01G017570*) (Figure 5a–5d). We then designed sgRNAs for each gene using the web tool at http://skl.scau.edu.cn [43] (Figure 5a–5d). Melon and cucumber share the same sgRNA1 at the conserved region of the first exon. The two homologous squash genes share the same sgRNA2 sequence in their conserved coding sequence (CDS) position. The sgRNA sequences were submitted to a BLAST search against CuGenDB, and no other targets were found. The sgRNAs were assembled into the binary vector *pBSE402* under the control of the *Arabidopsis* U6 promoter and the Cas9 coding sequence under the control of the cauliflower mosaic virus (CaMV) 35S promoter. Primers are shown in Table S2 (Figure 5e). The resulting constructs were then transformed into melon m1, squash jingxinzhen No. 4 and cucumber Cu2 using their respective optimized *Agrobacterium* transformation protocols.

To determine the mutations introduced by the CRISPR/Cas9 system in T_0_ plants, we amplified a PCR product overlapping with the target sequence for 3 melon T_0_ plants (*cmer_line2*, *cmer_line3*, and *cmer_line4*), 3 cucumber T_0_ plants (*cser_line3*, *cser_line4*, and *cser_line5*), and 2 squash T_0_ plants (*cmoer_line1* and *cmoer_line2*) and analyzed it using the high-throughput tracking of mutations (Hi-TOM) platform. Each individual T_0_ plant harbored multiple mutated alleles based on Hi-TOM sequencing data, highlighting the efficient mutation ratio of this CRISPR/Cas9 system in cucurbit crops. Mutations that occurred at target sites were considered to be “edited” based on the theory that Cas9 nuclease can cleave 3–5 bp upstream of the PAM site [44]. Plants with a target edited ratio greater than 1% (the number of reads with target mutations divided by the total number of reads of the target site) were regarded to be “edited” plants [45]. Sequencing data revealed that the editing frequency ranged from 23.47% to 40.69% in three independent T_0_ edited melon plants (Figure 5f) and 31.95% to 36.49% in three independent T_0_ edited cucumber plants (Figure 5g). The editing efficiency of two independent T_0_ squash plants at two loci was in the range of 18.94–25.78% and 22.66–39.49%, respectively (Figure 5h and 5i). The type and proportion of mutations in melon, squash, and cucumber T_0_ plants were also analyzed (Figure 5f–5j). The deletion or insertion size ranged from 1 to 9 bp in melon and cucumber, whereas more editing types and larger deletion sizes were obtained in edited squash plants. This result confirms that the CRISPR/Cas9 system efficiently edits cucurbit crop genomes.

To assess the off-target effects of the CRISPR/Cas9 system in cucurbit crops at the genome-wide level, whole-genome sequencing with a sequencing depth of 50× was performed on eight T_0_ edited cucurbit plants and three wild-type (WT) melon, cucumber, and squash plants. The ten most likely off-target sites (OT1–10) were selected for each sgRNA (Table 4) using Cas-OFFinder algorithms by comparing the sgRNA target to the reference genome (seed sequence mismatches ≤5 bp with the sgRNA sequences) as previously reported [46, 47]. To detect the mutations that occurred at potential off-target sites, including insertions, deletions, and substitutions, the sequence data were analyzed using the CRISPResso algorithm [48]. The genomes of edited plants were compared with those of WT plants to filter out genotypic and somaclonal background variation. WGS data indicated that there was only a low rate of base substitution mutations, and no indels were detected in edited plants, suggesting that no *bona fide* off-target mutations occurred at these potential off-target sites (Figure S6 and Table S3). These results suggest that the CRISPR/Cas9 system can precisely edit cucurbit crops with no off-target effects.

**Table 4:** The ten most likely potential off-target sites identified using Cas-OFFinder

**sgRNA**	**ID**	**Sequence**
Melon-sgRNA	OT1	TTCTaTcCTACCGTTGaTGAGG
	OT2	TTCTGTcCcACaGTTGGTGAGG
	OT3	TTCTGccCcACaGTTGGTGAGG
	OT4	TTtTGTGCTcCCtTTtGTGGGG
	OT5	cTCTtTGCTtCtGTTGGTGTGG
	OT6	gTCTGTcCTAtaGTTGGTGAGG
	OT7	TTCTGTctcACaGTTGGTGAGG
	OT8	TTtTGTGCgACatTTGGTGTGG
	OT9	TTaTGTGCTtaCtTTGGTGGGG
	OT10	TTaTtctCTACCGTTGGTGAGG
Cucmber-sgRNA	OT1	TTtTGTGCTtggGTTGGTGTGG
	OT2	cTCTtTGCTtCtGTTGGTGTGG
	OT3	TTCTaTGaTAtCGTaGGTGTGG
	OT4	TTCTGTGtTAaacTTGGTGTGG
	OT5	ggtTGTGCTACCGgTGGTGTGG
	OT6	TTCTcTaCaACtGTTGGTGTGG
	OT7	TTgTGTGtTAtCGTTGcTGTGG
	OT8	TTCTtTtCTACgaTTGGTGCGG
	OT9	TTtTGTGCTtCaGgTGGTGGGG
	OT10	TTCaGTGCaACaaTTGGTGTGG
Squash-sgRNA	OT1	cACAGTtACCAATCTCATCAGG
	OT2	cACAGTtACCAATCTCATCAGG
	OT3	AACAGcCACCAATaTCcTCAGG
	OT4	AACAGaCttCAATtTCATCAGG
	OT5	AACAGTtcCCAATacCATCAGG
	OT6	AAaAtctACCAATCTCATCCGG
	OT7	AAaAGTaACCgcTCTCATCTGG
	OT8	AACAtcCtCCAATtTCATCAGG
	OT9	AACAGcCACaAtTCaCATCTGG
	OT10	AACAaTaAtCcATCTCATCAGG

Lowercase letters represent mismatches with sgRNA.

### Creating heritable dwarf mutants in cucurbit crops

**Figure 6:**
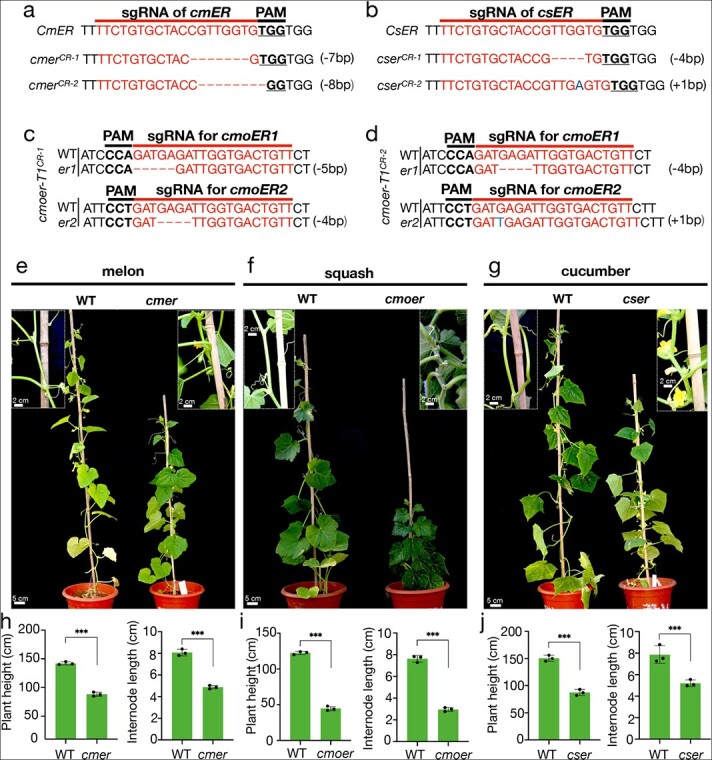
**Editing type and phenotype of homozygous mutants for the *ER* gene in the T**_**1**_  **generation.** (a, b, c and d) Different homozygous mutation types for the *ER* gene in T_1_ melon, cucumber, and squash, respectively. sgRNA and protospacer-adjacent motif (PAM) sequences are highlighted by red and bold, respectively. Red dash indicates deletion. Blue letters indicate insertion. (e, f, and g) Phenotypic observation of *er* mutants in the T_1_ generation with decreased height and short internodes in melon, squash, and cucumber. (h–j) Comparative analyses of plant height and internode length between wild type and mutants. Data were compared using a one-sided Student’s *t*-test. ^***^p < 0.01.

All T_0_ plants were self-pollinated, and the T_1_ progeny were analyzed to select Cas9-free plants with homozygous mutations using Sanger sequencing and GFP screening. Seeds without GFP (indicating no T-DNA insertion) were selected, and then Sanger sequencing was performed. Homozygous mutant T_1_ progeny were selected and verified by *Cas9* amplification. Homozygous mutagenesis of melon *ER* (*cmER*) resulted in null mutants with a 7-bp deletion and an 8-bp deletion (Figure 6a). Two null mutants of cucumber *ER* (*csER*) were also obtained, with a 4-bp deletion and a 1-bp insertion (Figure 6b). Homozygous double mutant materials of *cmoer1* and *cmoer2* were obtained from more than 1000 squash T_1_ progeny with null mutants (Figure 6c and 6d). Phenotypic analysis showed that the average plant height of the *cmer* null mutant was shorter than that of the WT by about 40%, and this was largely associated with shorter internodes (Figure 6e and 6h). Squash *cmoer* edited mutants had a more pronounced dwarf phenotype than the melon *cmer* mutant, with internodes being 60% shorter in the mutant than the WT (Figure 6f and 6i). Genome editing of *ER* homologs in cucumber also produced a similar phenotype, as internodes were 34% shorter in the *cser* mutant than in the WT (Figure 6g and 6j). These results demonstrated that homologous *ER* genes are responsible for internode elongation in cucurbit crops and that the generation of *er* mutants with short internodes may prove useful in breeding. To our knowledge, this study is the first to demonstrate stable knockouts using CRISPR/Cas9 genome editing technology in melon and squash.

## Discussion

The *Cucurbitaceae* family has long been recognized as one of the most difficult crops to transform, seriously hindering the application of genome editing tools. Successful genetic transformations and genome editing have been reported in cucumber and watermelon [13, 14]. However, the transformation efficiency of cucumber remains very low, and the indirect organogenesis used for watermelon regeneration is currently not compatible with other cucurbit crops. Therefore, other economically important *Cucurbitaceae* crops, such as melon and squash, remain difficult to stably transform, which greatly hinders functional genome research and the rapid creation of mutations that confer desirable traits in these crops.

*Agrobacterium* infiltration is thought to be the key to establishing an efficient and stable genetic transformation system. Adventitious shoots initiate from procambium or cambium cells in different plant species during organogenesis [25], and these comprise the target tissue for *Agrobacterium* infection. However, although previous studies recognized the need to infect the deeper cell layers of explants, no unified, standard protocol has been developed to achieve this goal. In practice, a fine balance must be struck between sufficient infection of competent cells and excessive infection of the entire explant, which would cause severe damage and reduce transformation efficiency.

To overcome these issues, we adopted an optimal intensity of infiltration strategy to identify the specific set of conditions that ensure complete infection of cambium cells in the vascular tissue while causing as little damage to explants as possible. Here, we determined the optimal intensity for vacuum infiltration, sonication, and micro-brushing to obtain good transformation efficiency for melon and squash, and we also added the antioxidant LA to the culture medium to protect explants from damage. Under these optimized conditions, vascular tissues were most effectively infected with the least damage to explants, resulting in stable genetic transformation of melon and squash with efficiencies of about 4%. Using the same strategy, we improved the transformation efficiency of different cucumber germplasms and partially overcame genotype dependence.

This efficient and stable genetic transformation system in cucurbit crops allowed us to perform CRISPR/Cas9-mediated genome editing in these species. The Arabidopsis *ER* gene family encodes leucine-rich repeat receptor-like kinases that have been shown to regulate stem elongation [27]. Editing cucurbit *ER* homologs produced *er* mutants in melon, squash, and cucumber. All mutants had shorter internodes and dwarf phenotypes, which may be advantageous for improving plant architecture and breeding.

To conclude, we have presented an efficient and stable genetic transformation system for melon and squash and demonstrated that CRISPR/Cas9-mediated genome editing can be effective in these cucurbit crops. The method described here will considerably accelerate research on cucurbit crops, both for basic plant biology and for the creation of elite lines.

## Materials and methods

### Media used in the study

GM (germination medium): 6-benzylaminopurine (BA, 2 mg/L for Cu2, 404 and Eu1, 0.5 mg/L for melon and Xintaimici, 1 mg/L for jingxinzhen No. 4 and jingxinzhen No. 11) and 1 mg/L abscisic acid (ABA) were added to MS medium.

IM (inoculation medium): BA (2 mg/L for Cu2, 404 and Eu1, 0.5 mg/L for melon and Xintaimici, 1 mg/L for jingxinzhen No. 4 and jingxinzhen No. 11), 1 mg/L ABA, 200 μM acetosyringone (AS) and 1.25 M morpholinoethanesulfonic acid (MES) (pH 5.2) were added to MS medium.

COM (co-cultivation medium): BA (as in IM, above), 1 mg/L ABA, 200 μM AS, 1.25 mM MES (pH 5.2), and 250 μM LA were added to MS medium.

SIM (shoot induction medium): BA (as in IM, above), 2 mg/L AgNO_3_, 1 mg/L ABA and 200 mg/L Timentin were added to MS medium.

RIM (root induction medium): 200 mg/L Timentin and 2 mg/L AgNO_3_ were added to MS medium.

LB (liquid medium for *Agrobacterium* culture): LB liquid medium with kanamycin (50 mg/L) and rifampicin (25 mg/L).

### Plant materials and growth conditions

Ten melon varieties were analyzed in this study: P147 (cultivated *melo*), ivf105 (cultivated *agrestis*), m1 (cultivated *melo*), m2 (cultivated *melo*), m3 (cultivated *melo*), m4 (cultivated *melo*), m5 (wild *melo*), m6 (cultivated *melo*), m7 (cultivated *agrestis*) and Jingyu (commercial hybrid), of which ivf105, and m1 to m7 were provided by Huai-song Wang of the Institute of Vegetables and Flowers (IVF), Chinese Academy of Agricultural Sciences. The seeds of cucumber inbred line Cu2 (South China type), Xintaimici (Northern China type), 404 (Northern China type) and Eu1 (European-type cucumber) were used in this study. Two commercial moschata materials, jinxinzhen No. 4 and jigxinzhen No. 11, were used in squash transformation. All tissue cultures were maintained in a culture room at the differentiation and rooting stages. After rooting, the regenerated plants were transferred into an incubator for about a week and planted in a greenhouse under a light regime of 16 h light/8 h dark at a temperature ranging from 18°C to 24°C.

It was necessary to germinate seeds to obtain explants, and the conditions for seed germination were as follows. Seeds were soaked in warm, distilled water at 50°C for 30 min, and the seed coat was removed using tweezers. Seeds were surface sterilized with 75% ethanol for 15 s and 1% sodium hypochlorite solution for 15 min, then rinsed six times in sterile distilled water. Sterilized seeds of melon, squash, and cucumber were spread on Petri dishes containing 7.5 mL sterilized water for 1–2 days at 28°C. Only the Cu2 seeds were spread on Petri dishes containing GM medium at 28°C.

### sgRNA design and plasmid construction

The sgRNA was designed using the CRISPR-GE web tool (http://skl.scau.edu.cn). The sgRNA was inserted into the pBSE402 vector as previously described [49]. In brief, a 50-μL mix containing 1.5 μL 100 μM forward primers, 1.5 μL 100 μM reverse primers, 5 μL 10× NEB buffer 3.1, and 42 μL water was incubated at 95°C for 5 min, and the temperature was then decreased by 0.1°C/s to 20°C to produce a short double-stranded DNA fragment. The short DNA fragment was inserted into pBSE402 using the restriction enzyme *Bsa*I and T4 Ligase (New England Biolabs). The constructed recombinant vector was transformed into *Agrobacterium* strain EHA105. Primers are shown in Table S2.

### *Agrobacterium*-mediated transformation

*Agrobacterium*-mediated transformation was performed as previously described with modifications [13]. Two days before transformation, *Agrobacterium* stock cells (EHA105) carrying the vector were shaken (200 rpm) using 3 mL liquid LB medium with 50 mg/L kanamycin and 25 mg/L rifampicin for 24 h at 28°C. The starter cultures were transferred to 50 mL liquid LB medium in a 1:1000 ratio and cultured overnight to OD_600_ 0.6–0.8. The *Agrobacterium* culture was centrifuged and resuspended to OD_600_ 0.2 with IM medium.

Germinated seeds (24–28 h for m1, Cu2, Xintaimici, 404 and Eu1, and 36 h for jinxinzhen No. 4) were transferred to a Petri dish that contained filter paper moistened with IM liquid medium, and the cotyledons were cut in half transversely. The hypocotyl was then removed, leaving a U-shaped cut end at the proximal of cotyledon. The cotyledon with a U-shaped cut end was used as the explant. Explants were scratched gently with a micro-brush in the region near the U-shaped cut end on the abaxial side 4–5 times along the direction from the distal axis to the proximal axis to ensure that the surface of the explant was wounded. Explants were always kept on wet filter paper. The treated explants were transferred to a 100-mL triangular bottle containing 20 mL *Agrobacterium* suspension. The bottle containing the explants and *Agrobacterium* suspension was sonicated at 100 W using an Ultrasonic cleaning instrument (KQ-100DV) different numbers of times (depending on the optimal infection conditions). During this period, the triangular bottle was gently shaken all the time to prevent the explants from sinking to the bottom. The explants were then transferred to a 20-mL medical syringe with 10 mL (for cucumber) or 15 mL (for melon and squash) *Agrobacterium* suspension. The air in the syringe was forced out, and the distal end of the syringe was sealed with a rubber cap. Slowly pulling the plunger from the 10-mL graduation line (cucumber) or 15-mL graduation line (melon and squash) to the 20-mL graduation line for 90 s (twice for cucumber, once for melon and squash) enabled the *Agrobacterium* to infiltrate the explant under vacuum. After the vacuum operation, the *Agrobacterium* suspension was discharged, and the explants were transferred to a Petri dish with dry filter paper. The liquid on the surface of the explants was removed, and the explants were cultured in the dark on COM solid medium with a piece of filter paper for 3 days. The GFP intensity of the explants after co-culture was used to judge the quality of transformation. The explants were rinsed with sterile water 5 to 6 times, then transferred to a 150-mm round glass dish with SIM; each dish contained 20 explants. To speed up bud regeneration, the explants were inserted into the medium slightly obliquely. The explants were cultured on SIM for 3–4 weeks. Explants with GFP fluorescent shoots were selected and transferred to jars containing RIM for rooting. Rooted seedlings were planted in the soil to promote growth and development. Many of these operations are difficult to describe clearly in words. For specific operations, please refer to Video S1.

### Detection of GFP fluorescence

To screen the GFP-positive plants, explants regenerated for one month were examined using a Leica MZ10 F stereomicroscope (Leica Microsystems, Germany) at the tissue culture stage. The GFP fluorescence of the plants was observed upon excitation with a LUYOR-3415RG light source (Luyor Instrument, Shanghai) in the greenhouse. The explants treated with different infection intensities were sectioned by hand along the transverse and longitudinal directions on ice, and GFP fluorescence of portions with vascular tissue was observed immediately under the stereomicroscope.

### GFP intensity calculation

The GFP expression level of the explants after co-cultivation was calculated using ImageJ as outlined in the protocol that follows. The fluorescence microscope is used to take an image. After adding the image to ImageJ, extract a single channel (Image-Color-Split Channels). Segment the channel first when the image is stored in RGB format; the threshold operation can be performed directly when the image is in 16-bit or 8-bit format. Then adjust the threshold and select the appropriate area (Image-Adjust-Threshold). The software will automatically select a default value; to eliminate the error caused by manually selecting the threshold value for different photos, it is best to use the default threshold value. Note that if there is a scale on the picture, it is necessary to adjust the threshold or select the Edit-Fill area of the scale to remove it; otherwise, it will affect the result. Use Red to characterize the selected area. The background of fluorescence images is usually black; therefore, check Dark Background. Next, select the appropriate threshold algorithm (Image-Adjust-Auto Threshold) in the Auto Threshold interface. Choose Try all here, and after clicking “OK”, all thresholds set by the algorithm will be listed. According to the result, select the Default algorithm. Next, set the parameters to be measured (Analyze-Set Measurements). Make sure to check Mean gray value and Limit to threshold, then click “OK”. Finally, the detection (Analyze-Measure) will produce the detection result after “Measure” is clicked: Mean is the average fluorescence intensity. Mean indicates the Mean gray value, equal to IntDen/Area, where IntDen = integrated density (sum of fluorescence intensity). Record the value of the sum of integrated intensity.

### Detection of targeted mutations

To detect the mutations that occurred at the target site, genomic DNA was extracted from all T_0_ plants and T_1_ plants using the CTAB method. PCR for target site amplification was performed using KOD-One (TOYOBO) and the primers listed in Table S2. To determine the mutations introduced by the CRISPR/Cas9 system, the PCR products amplified from different transgenic plants were cloned with the 5min TA/Blunt-Zero Cloning Kit (Vazyme Biotech). The positive colonies were sequenced, and sequences were aligned with WT genome sequences.

### Detection of editing efficiency with Hi-TOM sequencing

To determine the editing efficiency in T_0_ GFP-positive plants, Hi-TOM (high-throughput tracking of mutations) sequencing was performed as previously described [50]. The first-round PCR to amplify the targeted region was performed using site-specific primers with bridging sequences (5′-ggagtgagtacggtgtgc-3′ for the forward primer and 5′-gagttggatgctggatgg-3′ for the reverse primer) added at the 5′ end (primers listed in Table S2). The 25-μL reaction mix contained 60 ng genomic DNA, 0.4 μM specific primers, and 12.5 μL 2× GoTaq Green Master Mix (Promega). The second-round PCR was performed in a 20-μL volume with a pair of markers, and 4-bp barcode tags were separately added to the 5′ end of the primers for each sample; 1 μL primary PCR product was added as the template. The DNA products from the secondary amplification were sequenced on an Illumina sequencer, and the sequencing data were analyzed using the Hi-TOM website (http://www.hi-tom.net/hi-tom/).

### Off target analysis based on whole-genome resequencing (WGS)

Genomic DNA was extracted from fresh leaves of each T_0_ plant and WT for cucumber, melon, and squash using the CTAB method. The extracted DNA was sequenced using an MGI DNBSEQ-T7 sequencer with at least 50× coverage depth by Annoroad Gene Technology (Beijing). Potential off-target sites were identified using the Cas-OFFinder algorithms by comparing the sgRNA target sites to each reference genome [46, 47]. The sequenced data were analysed using the CRISPResso algorithm. The genome of edited plants was compared with the genome of WT plants to filter out background variation. The number of reads of various modification types was used to statistically calculate the mutation efficiency.

## Supplementary Material

Web_Material_uhab086

## Data Availability

Relevant data can be found within the paper and its supporting materials. All data from this study are available from the corresponding author upon reasonable request.
