# Supplementary material for: Targeted creation of new mutants with compact plant architecture using CRISPR/Cas9 genome editing by an optimized genetic transformation procedure in cucurbit plants
Source: Hortic Res. 2022 Jan 20;9:uhab086. doi: 10.1093/hr/uhab086 (PMC9016859; doi:10.1093/hr/uhab086)
Supplement: Web_Material_uhab086 [file web_material_uhab086.zip › Supplemental Figures.docx]

**Supplemental Figures**


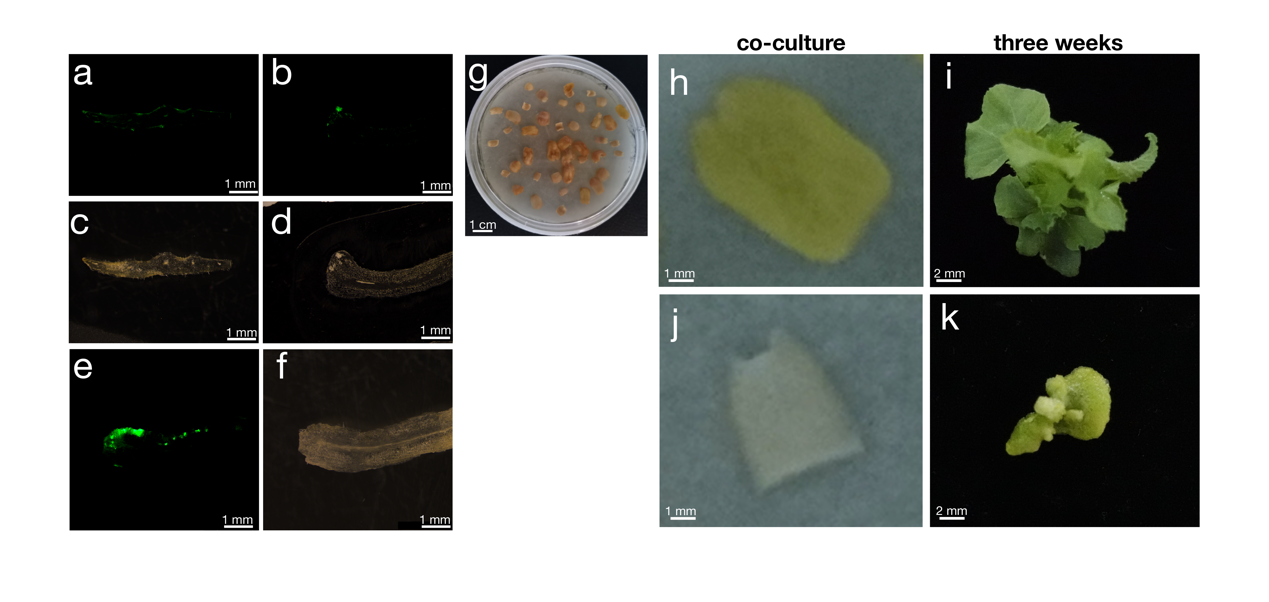


**Figure S1. Optimization of genetic transformation system of melon**

(a, b, c and d) Cambium cells located in the deep layer of vascular tissue could not be infected using the vacuum assisted method published by *Hu et al*. The images (a and b) were taken under the GFP channel. The image (c and d) shows the same shoot in the bright field.

(e and f) Vacuum treatment with longer duration can increase the expression of GFP, but the vascular tissue in the deep layer cannot be infected.

(g) Most explants were dead with longer duration vacuum.

(h and k) The health explant and regenerated shoot, and (j and k) the damaged explants could not regenerate into buds.


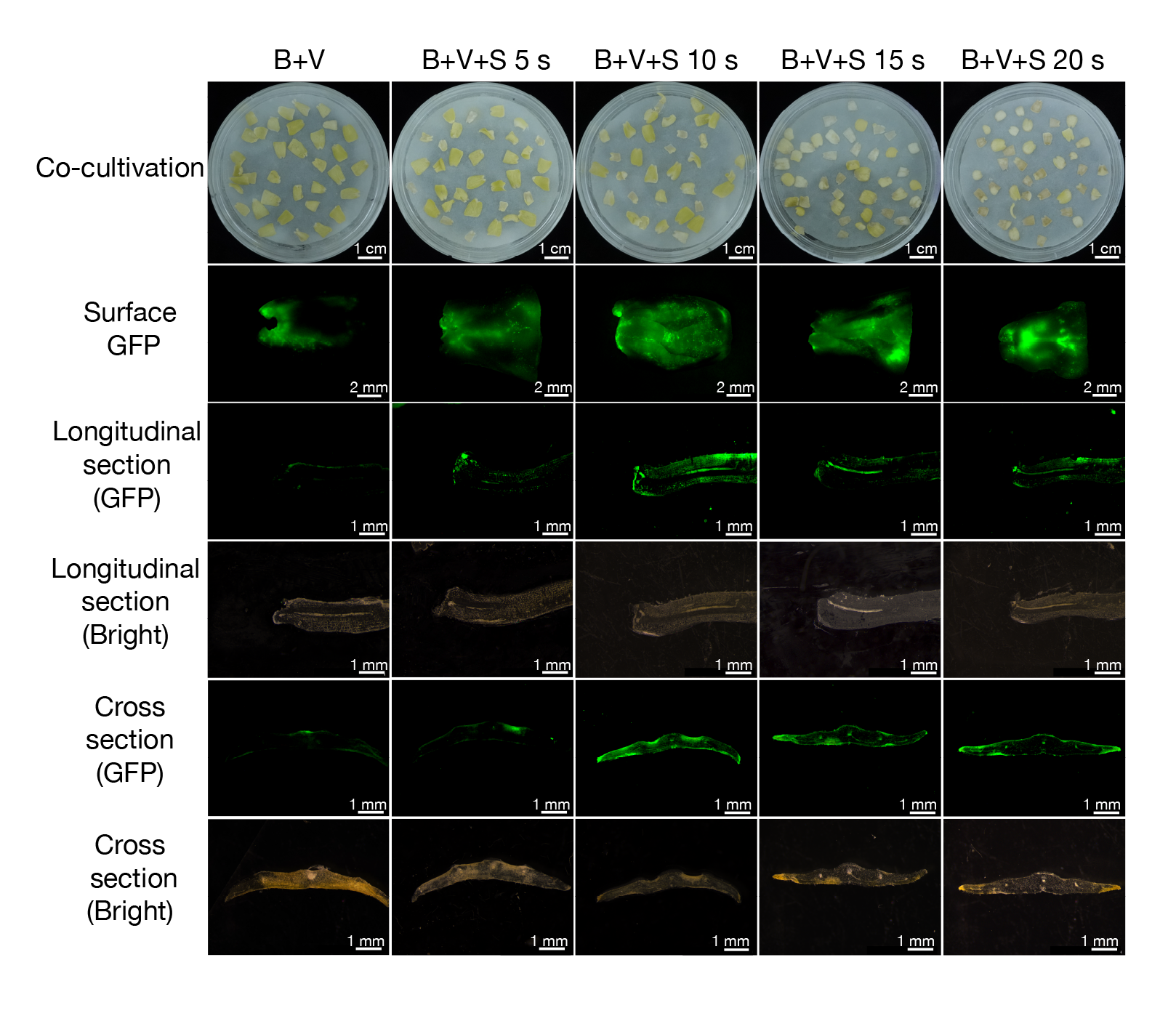


**Figure S2. Optimal infiltration intensity test for melon m1.**

From left to right, each column represents different infection treatments and effects. V: vacuum, B: brush, S: sonication. The first line represents cotyledonary explants after co-cultivation with *Agrobacterium tumefaciens*; from the second line to the six line, examination of GFP fluorescence after co-cultivation showed that the region and intensity of the fluorescent signal; the third and the fourth line represent the infected areas of longitudinal section; the fifth and the sixth line represent the infected areas of cross-section; The white solid wireframe represents an enlarged view of the vascular bundle tissue location.


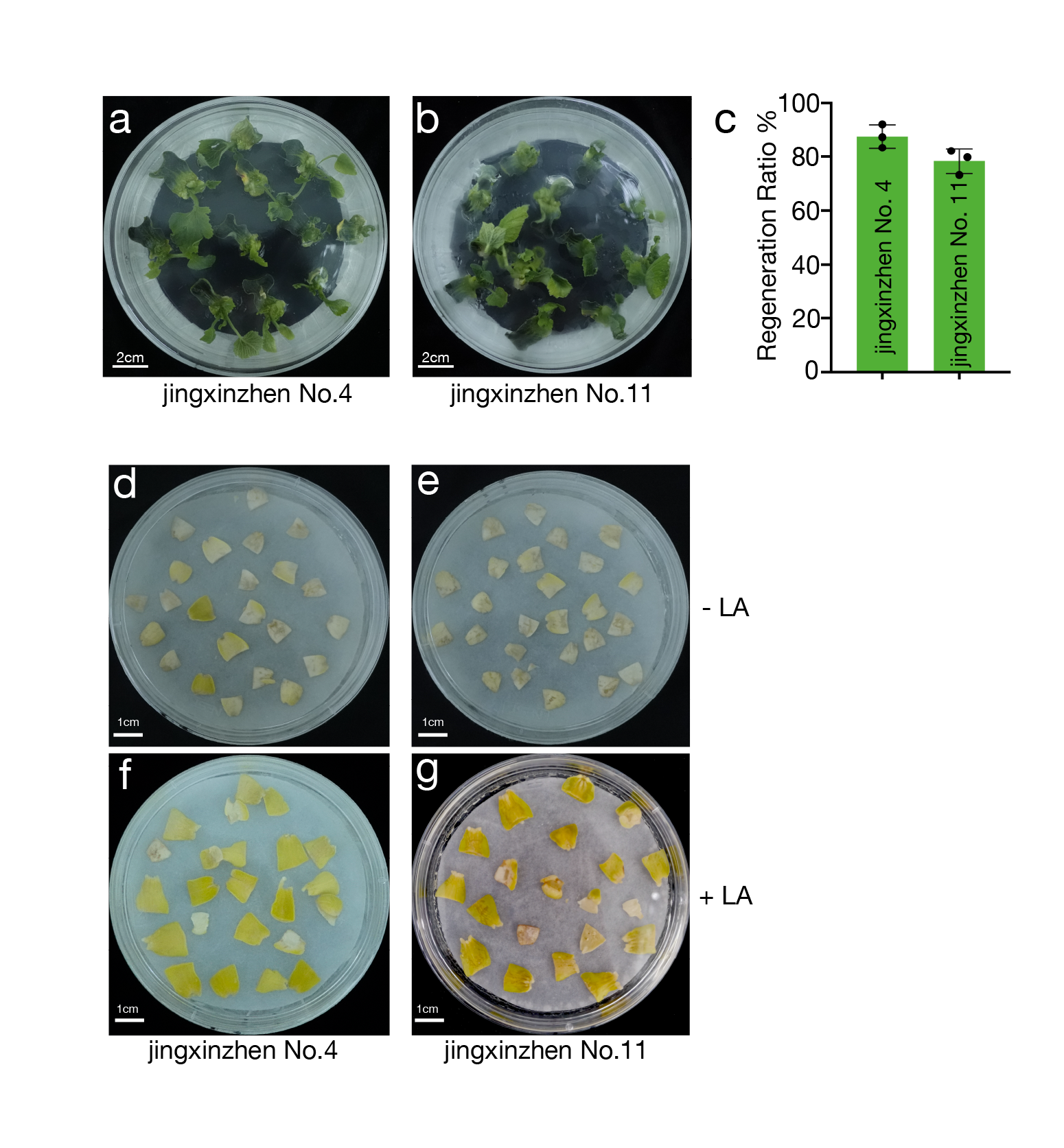


**Figure S3. The explant regeneration of squash and antioxidant LA have protective effects on explants.**

(a-c) Comparison of regeneration ratio of jingxinzhen No.4 and jingxinzhen No.11.

(d and e) The explants of jingxinzhen No.4 and jingxinzhen No.11 turned white and died under the condition of sonication for 5 s.

(f and g) The explants of jingxinzhen No.4 and jingxinzhen No.11 after co-culture under the condition of sonication for 5 s in the presence of LA.


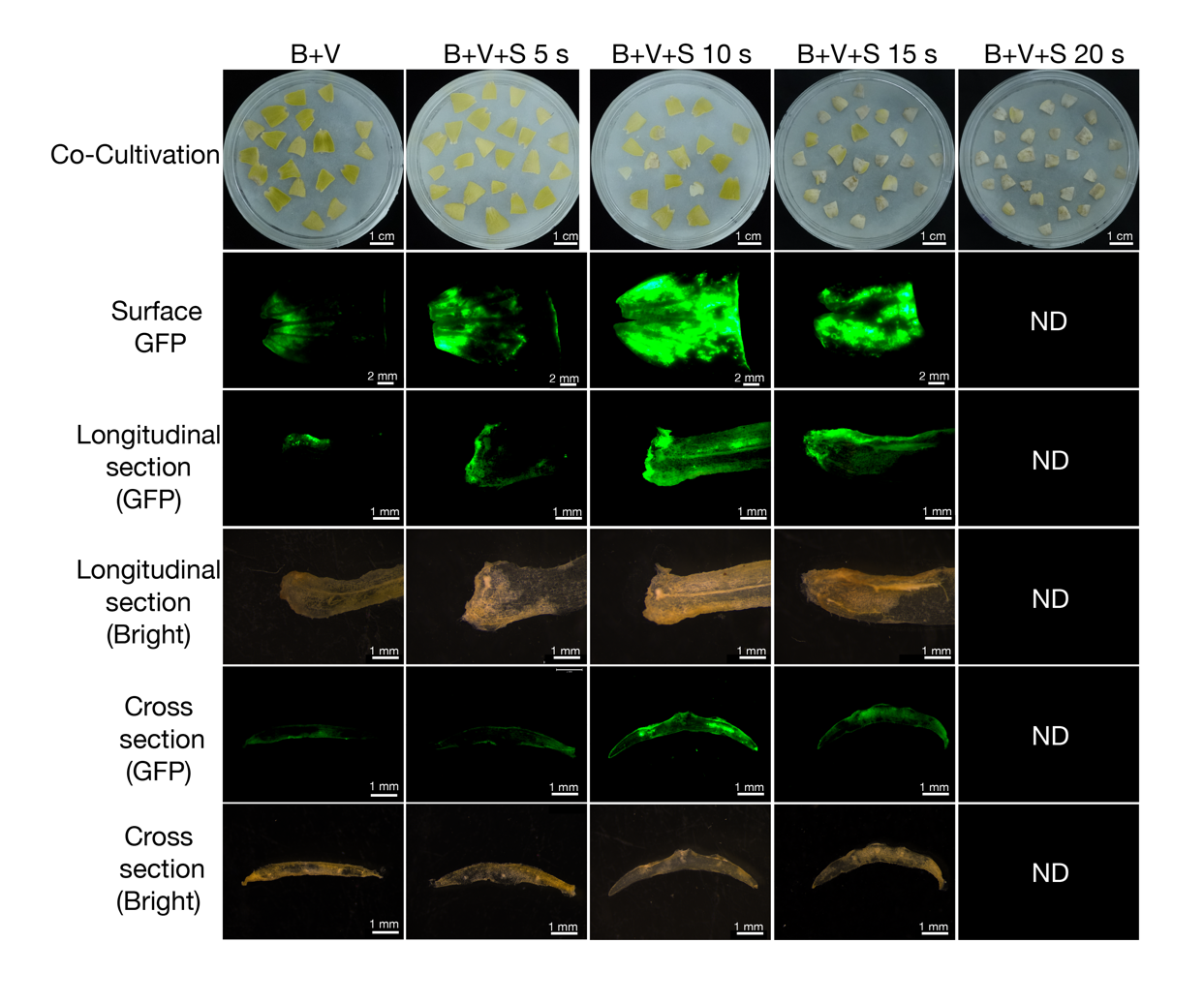


**Figure S4. Optimal infiltration intensity test for squash jingxinzhen NO.4.**

From left to right, each column represents different infection treatments and effects. V: vacuum, B: brush, S: sonication. The first line represents cotyledonary explants after co-cultivation with *Agrobacterium tumefaciens*; from the second line to the six line, examination of GFP fluorescence after co-cultivation showed that the region and intensity of the fluorescent signal; the third and the fourth line represent the infected areas of longitudinal section; the fifth and the sixth line represent the infected areas of cross-section; The white solid wireframe represents an enlarged view of the vascular bundle tissue location.


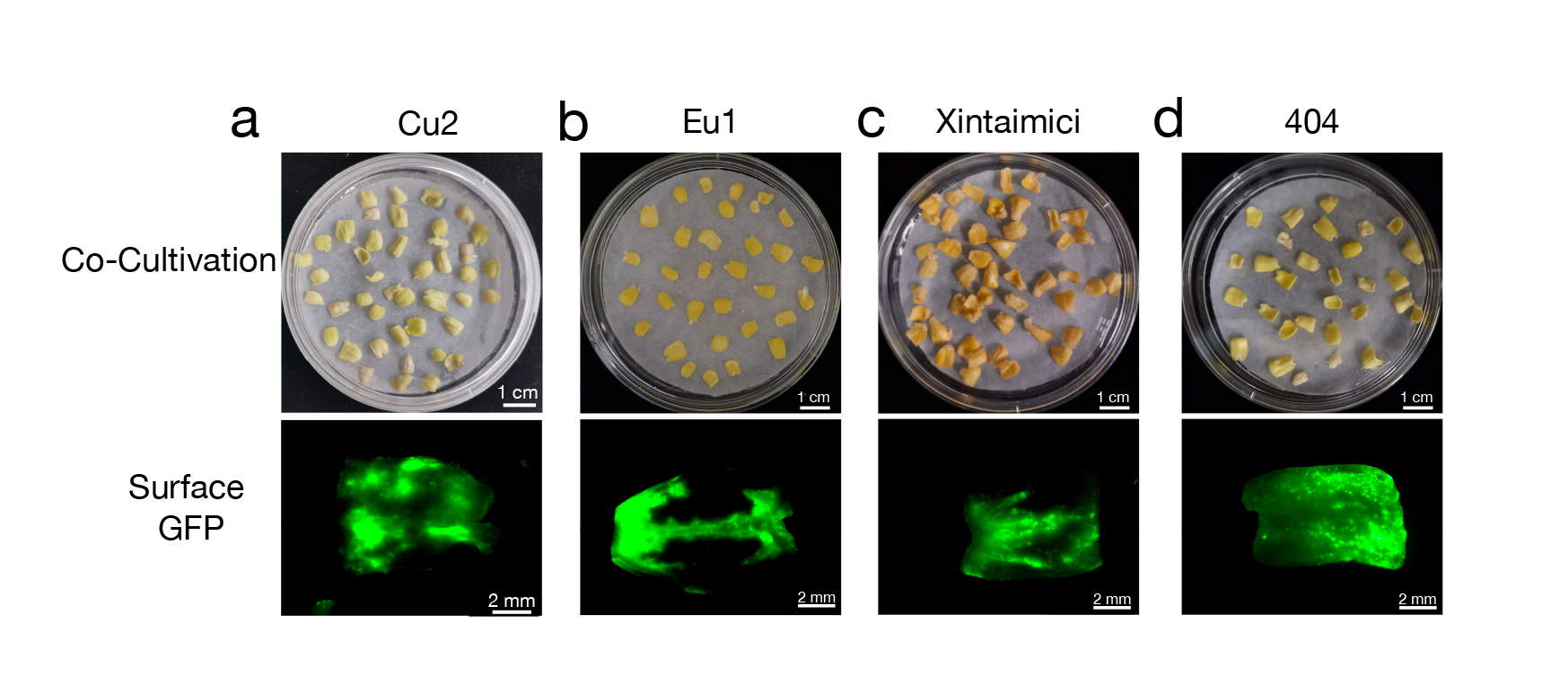


**Figure S5. The optimal infiltration intensity of different cucumber germplasms.**

(a-d) From left to right, each column represents different cucumber germplasm. The first line represents cotyledon explants of melon after co-cultivation with *Agrobacterium tumefaciens* in the dark, from the second line to the sixth line, examination of GFP fluorescence after co-cultivation showed that the region and intensity of the fluorescent signal.


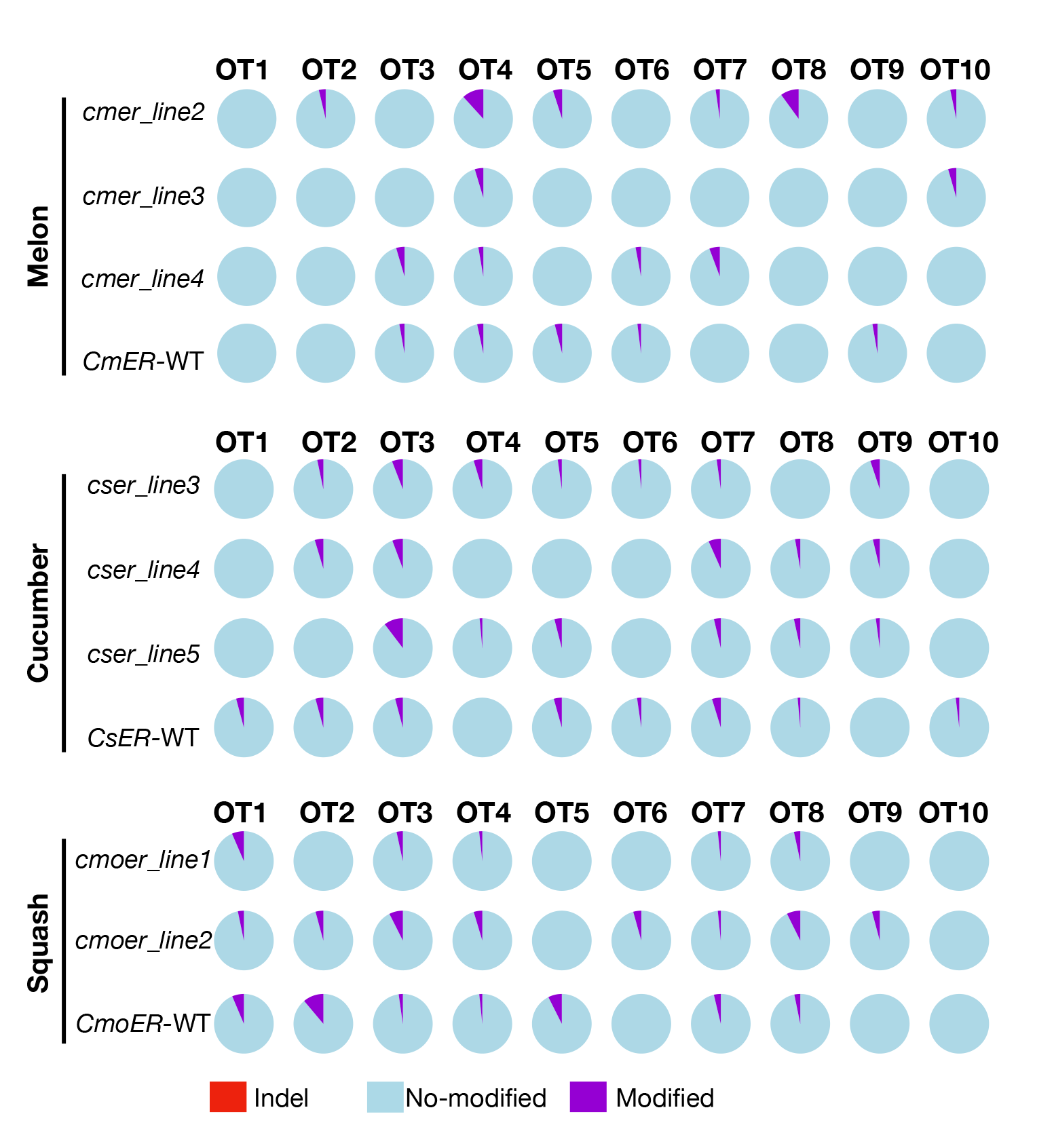


**Figure S6. The evaluation of off-target effects by whole-genome sequencing (WGS).**

The mutation patterns were detected in 10 most likely potential off-target sites for each sgRNA in melon, cucumber and squash. Every pie chart represents mutation type of each site for one line.
