# Supplementary material for: Targeted creation of new mutants with compact plant architecture using CRISPR/Cas9 genome editing by an optimized genetic transformation procedure in cucurbit plants
Source: Hortic Res. 2022 Jan 20;9:uhab086. doi: 10.1093/hr/uhab086 (PMC9016859; doi:10.1093/hr/uhab086)
Supplement: Web_Material_uhab086 [file web_material_uhab086.zip › Supplemental Tables.pdf]

Table S1. Shoot regeneration ratio of ten different melon varieties.

	P147			ivf105			m7			m6			m5		
	No. of regeneration	No. of explants	Ratio	No. of regeneration	No. of explants	Ratio	No. of regeneration	No. of explants	Ratio	No. of regeneration	No. of explants	Ratio	No. of regeneration	No. of explants	Ratio
rep1	81	96	0.84375	58	72	0.8056	84	93	0.903	4	98	0.041	5	98	0.051
rep2	83	103	0.80583	65	83	0.7831	87	99	0.879	3	64	0.047	2	48	0.042
rep3	92	114	0.80702	73	92	0.7935	72	84	0.857	3	72	0.042	3	72	0.042

	m4			m3			m2			m1			jingyu		
	No. of regeneration	No. of explants	Ratio	No. of regeneration	No. of explants	Ratio	No. of regeneration	No. of explants	Ratio	No. of regeneration	No. of explants	Ratio	No. of regeneration	No. of explants	Ratio
rep1	23	104	0.22115	72	104	0.6923	77	93	0.828	104	112	0.929	67	82	0.817
rep2	35	112	0.3125	54	93	0.5806	82	103	0.796	89	93	0.957	72	93	0.774
rep3	23	92	0.25	62	98	0.6327	65	90	0.722	78	84	0.929	58	84	0.69

Table S2. Primers used in this study.

Primer name	Sequence (5'-3')
**Primers for construction of gRNA expressing vectors targeting specific genes**	
Cri-cuc-ER-F	ATTGTTCTGTGCTACCGTTGGTG
Cri-cuc-ER-R	AAACCACCAACGGTAGCACAGAA
Cri-melon-ER-F	ATTGTTCTGTGCTACCGTTGGTG
Cri-melon-ER-R	AAACCACCAACGGTAGCACAGAA
Cri-mosach-ER-F	ATTGAACAGTCACCAATCTCATC
Cri-mosach-ER-R	AAACGATGAGATTGGTGACTGTT
U626-IDF	TGTCCCAGGATTAGAATGATTAGGC
**Primers for analysis of target gene mutations**	
cuc-ER-F	ATGAAGTCTGTGAAGAGGGCAG
cuc-ER-R	CTCCTAGAGTTGAGAATAGAAG
Melo-ER-jc-F	TCCCATTACAGCCTCACAGCAT
Melo-ER-jc-R	CATGTAACTAAAGATCTTAGCTC
Mosch-ER1-JC-F	CTCCTTCTGTCGGCAACCTCA
Mosch-ER1-JC-R	TTAGTATGTCACTGTGTAAGTC
Mosch-ER2-JC-F	TCTGGTCTCAACCTGGTCGGAGA
Mosch-ER2-JC-R	TTCTATATGATTGGACAAGCTC
**Primers for Hi-Tom**	
Hi-Tom-csER-F	GGAGTGAGTACGGTGTGCATGAAGTCTGTGAAGAGGGCAG
Hi-Tom-csER-R	GAGTTGGATGCTGGATGGCATTCGTCCTGATTTGATTACC
Hi-Tom-melonER-F	GGAGTGAGTACGGTGTGCATGAAGTATGTGAAGAGGGCAG
Hi-Tom-melonER-R	GAGTTGGATGCTGGATGGCATTCCTCCTGATTTGATTACC
Hi-Tom-moschER1-F	GGAGTGAGTACGGTGTGCTTACATGGCTGAGTGGTGTTC
Hi-Tom-moschnER1-R	GAGTTGGATGCTGGATGGGGCTAACCGTGAGCTTAAGCCA
Hi-Tom-moschER2-F	GGAGTGAGTACGGTGTGCTTCATGTTCTTACTTTCTAAGCC
Hi-Tom-moschnER2-R	GAGTTGGATGCTGGATGGGAAGATATCGAACATTTGTAAGC

Table S3. Detection of the potential off-target sites by whole-genome sequencing

lines	Mutation	Mutation rate (%)									
		OT1	OT2	OT3	OT4	OT5	OT6	OT7	OT8	OT9	OT10
cmer_line2	Indel	0.00	0.00	0.00	0.00	0.00	0.00	0.00	0.00	0.00	0.00
	Modified	0.00	3.70	0.00	11.76	5.00	0.00	2.27	10.00	0.00	3.28
cmer_line3	Indel	0.00	0.00	0.00	0.00	0.00	0.00	0.00	0.00	0.00	0.00
	Modified	0.00	0.00	0.00	4.65	0.00	0.00	0.00	0.00	0.00	4.44
cmer_line4	Indel	0.00	0.00	0.00	0.00	0.00	0.00	0.00	0.00	0.00	0.00
	Modified	0.00	0.00	4.55	2.70	0.00	2.94	5.71	0.00	0.00	0.00
cmer_WT	Indel	0.00	0.00	0.00	0.00	0.00	0.00	0.00	0.00	0.00	0.00
	Modified	0.00	0.00	2.78	3.33	4.00	1.92	0.00	0.00	2.70	0.00
cser_line3	Indel	0.00	0.00	0.00	0.00	0.00	0.00	0.00	0.00	0.00	0.00
	Modified	0.00	3.28	5.88	4.69	2.17	1.67	2.22	0.00	5.13	0.00
cser_line4	Indel	0.00	0.00	0.00	0.00	0.00	0.00	0.00	0.00	0.00	0.00
	Modified	0.00	4.65	5.71	0.00	0.00	0.00	6.67	2.82	3.70	0.00
cser_line5	Indel	0.00	0.00	0.00	0.00	0.00	0.00	0.00	0.00	0.00	0.00
	Modified	0.00	0.00	10.34	1.45	4.00	0.00	3.70	3.45	2.13	0.00
cser_WT	Indel	0.00	0.00	0.00	0.00	0.00	0.00	0.00	0.00	0.00	0.00
	Modified	4.17	4.26	4.17	0.00	4.35	2.27	4.76	1.43	0.00	1.89
cmoer_line1	Indel	0.00	0.00	0.00	0.00	0.00	0.00	0.00	0.00	0.00	0.00
	Modified	6.49	0.00	3.39	1.69	0.00	0.00	1.61	3.41	0.00	0.00
cmoer_line2	Indel	0.00	0.00	0.00	0.00	0.00	0.00	0.00	0.00	0.00	0.00
	Modified	3.23	4.23	7.41	4.65	0.00	4.35	1.59	7.32	4.17	0.00
cmoer_WT	Indel	0.00	0.00	0.00	0.00	0.00	0.00	0.00	0.00	0.00	0.00
	Modified	6.35	11.11	2.22	1.64	7.41	0.00	3.70	3.17	0.00	0.00

Modified: base subsitution mutations, Indel: deletion and insertion.
